# Youth Sport and COVID-19: Contextual, Methodological, and Practical Considerations

**DOI:** 10.3389/fspor.2020.584252

**Published:** 2020-10-09

**Authors:** Adam L. Kelly, Karl Erickson, Scott Pierce, Jennifer Turnnidge

**Affiliations:** ^1^Department of Sport and Exercise, Research Centre for Life and Sport Sciences (CLaSS), School of Health Sciences, Birmingham City University, Birmingham, United Kingdom; ^2^Department of Kinesiology, Institute for the Study of Youth Sports, Michigan State University, East Lansing, MI, United States; ^3^School of Kinesiology and Recreation, Illinois State University, Normal, IL, United States; ^4^PLAYS Research Group, School of Kinesiology and Health Studies, Queen's University, Kingston, ON, Canada

**Keywords:** coronavirus, pandemic, athlete development, positive youth development, sports coaching, sport experiences

## Introduction

One of the growing concerns among youth sport stakeholders is how the COVID-19 pandemic will shape youth sport development. Given the novelty and rapidly changing nature of these events, the impacts on development are not yet clear. Thus, to gain a deeper understanding of the impact of this crisis, it is crucial for researchers and practitioners to examine the effects on youth development at different timescales. Although we are seeing immediate changes in the activities, social dynamics, and settings that are influencing youth's real-time experiences, questions remain regarding its influence on short-, and long-term developmental outcomes (Kelly et al., 2020). Moving forward, we will need to be cognizant of how this watershed moment will shape youth sport development for months and years to come. As such, this opinion article will focus on exploring potential contextual, methodological, and practical considerations that may be relevant as we navigate through these uncertain times. Further, we hope to encourage researchers and practitioners to embrace this as an opportunity to critically reflect and evaluate the existing practice of youth sport.

## Contextual Considerations

Since sport is an activity involving skill and physical exertion, whereby an individual or team gather to compete against each other for the purpose of enjoyment and performance, this moment is perhaps the first-time key stakeholders in youth sport have been required to engage in virtual provisions. Through this shift from face-to-face to virtual sport, we have also gained a new perspective of our social dynamics: the colliding of our sport and personal selves. Coaches, parents, and athletes are now seeing each other's homes, families, and pets in an unprecedented manner. We are also seeing the tensions that can arise from the collision of these multiple roles. As researchers and practitioners, we need to recognize that tensions between these roles are inevitable and that they are the result of an important fact: youth sport coaches, officials, administrators, parents, and athletes (amongst others) are all people beyond the sport environment.

It is thus more important than ever to adopt a person-centered approach, in which we recognize and value the roles and responsibilities of youth sport stakeholders beyond the sport context. At the centerpiece of this person-centered approach lies the athlete-centered focus on the child and their development. Refining and redefining social dynamics in youth sport provides an opportunity for us to ensure that interpersonal adult actions and interactions support the growth and experience of youth. It would thus be beneficial to document and share the ways key stakeholders are adapting during this time (e.g., supporting youth-led activities, aiding with technology and virtual sport sessions, researching strategies to safely engage in sport activities), so that we can continue to foster quality relationships between parents, coaches, and youth sport organizations.

One of the unique aspects of COVID-19 is its global scale. Youth sport around the world is being affected by this pandemic. However, each person's experiences are shaped by several contextual factors (e.g., demographics, country, cultural norms). For example, different countries (e.g., the UK vs. the USA vs. Sweden vs. South Korea), experienced different degrees of lockdown and accompanying restrictions on youth sport and physical activity, different levels of popular support for social distancing measures, as well as different timescales and protocols for return to play. Furthermore, although sport is a popular activity among youth all over the world, organizational structures can vary from country to country depending on the resources that are available (e.g., human, natural, and capital). As such, we need to ensure our research, programmes, and policies reflect this diverse representation of experiences and cultures.

A range of socioeconomic and cultural inequalities have been magnified during the COVID-19 pandemic (Evans et al., 2020). For example, Power et al. (2020) explain how COVID-19 has worsened inequalities between privileged and disadvantaged groups in the UK system of food supply and distribution. Furthermore, Laster Pirtle (2020) illustrates how racial capitalism is a fundamental cause of the ethnic and socioeconomic inequities presented during the COVID-19 pandemic in the USA. More specifically, the author uses the over-representation of black deaths reported in Detroit, Michigan, due to COVID-19 to portray the racial inequalities that are embedded throughout our societies.

As such, we also need to acknowledge how this pandemic will disproportionately affect segments of the sport community. Indeed, this crisis has shined a light on several of the cracks that have always existed in the sport system. Inequities based on gender, age, socioeconomic status, and level of ability, have all been exacerbated by this situation. As such, there is a significant risk for “Matthew effects” (Merton, 1968), which relates to the common notion of “the rich get richer and the poor get poorer.” These effects highlight that those with initial advantages based on gender, age, socioeconomic status, and level of ability, may be similarly advantaged during this crisis. For example, families with higher levels of income and job security may be more likely to live and work in settings that are conducive to both physical distancing and sport participation. These families may also be better able to withstand the detrimental effects of COVID-19 on the global economy and may be poised to re-engage with the youth sport system when sports resume. Thus, as we navigate this crisis, it will be important to ensure that steps are being taken to account for and address the inequities in our sport communities.

## Methodological Considerations

To develop programmes and policies to effectively facilitate positive outcomes during these unique circumstances, it will be crucial for researchers and practitioners to work together. The effects of this pandemic are being experienced in real-time. As such, researchers and practitioners will need to find creative ways to engage with each other to ensure that we are getting the right information, to the right people, in the right format, at the right time. To achieve this, we can embrace new ways of collaboration, such as harmonized data collection and rapid approaches to knowledge development, synthesis, and dissemination. We can also embrace the value of diverse perspectives and methodological approaches. The number of articles being published on COVID-19 is growing each day. However, we need to find ways of applying that knowledge in real-world settings. Finding ways to quickly synthesize these findings into practical tools that can be used to improve the quality of youth sport will be pivotal. As we move forward, it will be important for researchers and practitioners to develop new ways of capturing the immediate, short-, and long-term effects of this pandemic.

To sufficiently enhance our understanding of the impact of COVID-19 on youth sport development during these timescales (e.g., immediate, short-, long-term), the access to and feedback from key stakeholders (e.g., athletes, parents, coaches, administrators) employed in real-word settings is crucial. For instance, although we subjectively offer key considerations, without the knowledge and collaboration with sports clubs and organizations, the implications of COVID-19 on youth sport with remain inconclusive. Further, since COVID-19 has affected youth sport on a global scale, extensive participant recruitment for large sample sizes will also be required to offer a broad representation and not presume individuals as homogeneous. A potential solution to this issue is the creation of international research groups to explore the implications of COVID-19 on youth sport.

Indeed, in light of this, the authors have collectively begun to collaborate with multiple sports clubs and organizations across the world, to generate a large database for the impending “(Re)Imagining Youth Sport: The COVID-19 Lockdown” project (BCU, 2020). This project is a 3-phase examination of the current and future effects of the COVID-19 pandemic on youth sports in society, targeted at youth sport parents, coaches, and administrators worldwide. Phase 1 is currently underway and involves an online international survey of three key stakeholder groups (i.e., youth sport parents, coaches, and administrators; currently at ~500 responses from 18 countries across six continents). Phase 2 involves virtual one-on-one qualitative interviews with all three stakeholder groups to explore survey participants' responses in greater depth. Phase 3 will involve a 6-month follow-up online survey to examine participants' experiences as youth sport is gradually re-introduced. Taken together, the three phases of the project are framed as a practically-oriented needs assessment to inform future real-world knowledge mobilization efforts by youth sport organizations and their supporting communities. To this end, the project attempts to address two guiding research questions:

1. How are youth sport stakeholders experiencing the removal of Organized youth sports due to the COVID-19 pandemic?
2. How, and to what degree (if at all), do these stakeholders want and need youth sports to be different when they return after the pandemic?

Utilizing a range of survey and interview approaches, this study was designed not only to gather and examine multiple perspectives on the past and future of youth sport but also to prompt parents, coaches, and administrators to leverage this opportunity to reflect on, reimagine, and plan for a “new and improved” youth sport experience. As such, the authors welcome any interest from parties around the world wishing to collaborate and enhance this database further in pursuit of this goal. We would also like to acknowledge a number of other researchers are similarly exploring related issues. For example, in the USA (though not an exhaustive list), Dr Jennifer Agans at Pennsylvania State University (Vest Ettekal and Agans, 2020), Dr Tim McGuine at the University of Wisconsin (WSN, 2020), and Dr Travis Dorsch at Utah State University (Project Play, 2020), are all leading collaborative research projects examining aspects of COVID-19 and youth sport.

## Practical Considerations

Return to play provisions are at the top of organizational agendas during this unprecedented time (Pierce et al., 2020). As such, developing a safe return is paramount in the immediate future to ensure young athletes are not exposed to the spread of COVID-19 (see, for example, Department for Digital Culture Media Sport, 2020). Indeed, by feeling safe, sport environments can promote exploration, creativity, and a sense of security (Lerner et al., 2000; NRCIM, 2002). In addition, when sport was forced to shut down, we were faced with an uncomfortable reality. While beloved by millions, sport is not “essential.” Perhaps being confronted with this truth may enable us to keep sport in perspective. Safety and family do not have to be incompatible with sport success. We thus must strive to create better activities, social dynamics, and settings that foster all three to facilitate greater positive youth development outcomes.

As highlighted throughout this opinion, COVID-19 will have important implications for nearly every facet of the youth sport system, with changes to sport system becoming inevitable. It is also important to acknowledge that these changes may carry significant practical, financial, and health costs for youth sport stakeholders. As such, programmes and policies should focus on minimizing these secondary effects of this crisis and ensure we are creating safe and equitable youth sport environments. To achieve this goal, it may be useful to draw upon existing evidence-based recommendations for facilitating development in sport. In 2016, Côté and Hancock proposed 10 recommendations for designing youth sport programmes (Côté and Hancock, 2016). These recommendations included prioritizing sport activities that emphasize fun and short-term rewards, facilitating play-based and youth-led activities, and limiting the length of season and travel requirements. By drawing upon such recommendations, researchers, and practitioners can develop programmes and policies that will be relevant and beneficial both now and in the future.

Interestingly, many of these recommendations may be applicable within the COVID-19 context. For instance, this crisis offers an opportunity to shift our focus from practice-based, adult-led activities to play-based, youth-led activities, as well as restricting travel and promoting a cost effective approach, to focus on the quality of youth's immediate sport experiences. Previous research suggests that an increased accumulation of youth-led activities, such as deliberate play, can positively influence athletes' long-term participation, performance, and personal development (e.g., Côté and Erickson, 2015). Furthermore, youth-directed, practice-oriented activities, such as “spontaneous practice” in which young people independently engage in self-directed sport activities with skill improvement as the primary objective, may be particularly salient under lockdown conditions (Kelly et al., 2020).

In an ideal world, members of the youth sport community will continue to engage in youth sport throughout this crisis and into the future (Drummond et al., 2020). However, we need to acknowledge the potential challenges and difficulties of this crisis. For example, recent studies highlight how fears and uncertainty regarding COVID-19 can negatively influence psychological well-being, including anxiety, depression, and social isolation (e.g., BCU, 2020; ESPN, 2020; WSN, 2020). Further, our motivation or opportunity to participate in youth sport may be negatively affected by COVID-19. Because of this crisis, youth sport athletes, coaches, parents, and organizations may need to step back or dropout of sport for a time. Thus, throughout the lifecycle of this pandemic, we need to provide support and opportunities for individuals to get involved or re-engage with youth sport in the immediate, short-, and long-term. By providing such opportunities, we can positively influence the development of current and future youth sport participants.

## Conclusion

The unique circumstances surrounding COVID-19 offer an opportunity to reflect on existing youth sport provision. What defines a “real” sport? What are those key ingredients needed for sport to occur? Sport systems can often be resistant to change because this “is how we've always done it.” Now is the time to challenge that approach. Sports are not naturally occurring phenomenon—they are created by people. As such, they can be changed by people. We can choose to adapt sports to meet the needs of those who want to participate. More specifically, we can change what and how we engage in youth sport activities, how we interact with peers, coaches, parents, and communities, and the environments where we engage in sport. This leads us to perhaps the most important question: if we cannot make these changes now, then when?

## Author Contributions

AK and JT wrote the opinion article with the assistance of KE and SP. All authors contributed to the article and approved the submitted version.

## Conflict of Interest

The authors declare that the research was conducted in the absence of any commercial or financial relationships that could be construed as a potential conflict of interest.

## References

[B1] BCU (2020). Not Just a Game: Nearly 80 Percent Of Parents Report Decrease in Children's Wellbeing Due to Lack of Youth Sport During Lockdown. Available online at: https://www.bcu.ac.uk/about-us/coronavirus-information/news/not-just-a-game-nearly-80-per-cent-of-parents-report-decrease-in-childrens-wellbeing-due-to-lack-of-youth-sport-during-lockdown (accessed July 15, 2020).

[B2] Côté J. Erickson K. (2015). Diversification and deliberate play during the sampling years, in The Handbook of Sport Expertise, eds J. Baker and D. Farrow (London: Routledge), 305–16. 10.4324/9781315776675-27

[B3] Côté J. Hancock D. (2016). Evidence-based policies for youth sport programs. Int. J. Sport Pol. Pol. 8, 51–65. 10.1080/19406940.2014.919338

[B4] Department for Digital Culture Media Sport (2020). Return to Recreational Team Sport Framework. Retrieved from: https://www.gov.uk/government/publications/coronavirus-covid-19-guidance-on-phased-return-of-sport-and-recreation/return-to-recreational-team-sport-framework (accessed July 15, 2020).

[B5] Drummond M. Elliott S. Drummond C. Prichard I. (2020). Youth sport and COVID-19: A potential generation lost. Emerald Open Res. 2, 1–7. 10.35241/emeraldopenres.13661.1

[B6] ESPN (2020). Coronavirus Fears Spiking Anxiety, Depression in Professional Footballers – FIFPro Study. Retrieved from: https://www.espn.co.uk/football/fifa-world-cup/story/4085559/coronavirus-fears-spiking-anxietydepression-in-professional-footballers-fifpro-study (accessed July 15, 2020).

[B7] Evans A. B. Blackwell J. Dolan P. Fahlén J. Hoekman R. Lenneis V. (2020). Sport in the face of the COVID-19 pandemic: towards an agenda for research in the sociology of sport. Eur J Sport Soc. 17, 85–95. 10.1080/16138171.2020.1765100

[B8] Kelly A. L. Erickson K. Turnnidge J. (2020). Youth sport in the time of COVID-19: Considerations for researchers and practitioners. Manag. Sport Leisure. 1–11. 10.1080/23750472.2020.1788975. [Epub ahead of print].

[B9] Laster Pirtle W. N. (2020). Racial capitalism: a fundamental cause of novel coronavirus (COVID-19) pandemic inequities in the United States. Health Educ. Behav. 47, 504–508. 10.1177/1090198120922942. [Epub ahead of print]. 32338071 PMC7301291

[B10] Lerner R. M. Fisher C. B. Weinberg R. A. (2000). Toward a science for and of the people: Promoting civil society through the application of developmental science. Child Develop. 71, 11–20. 10.1111/1467-8624.00113 10836553

[B11] Merton R. K. (1968). The Matthew effect in science: The reward and communication systems of science are considered. Science 159, 56–63. 10.1126/science.159.3810.56 5634379

[B12] NRCIM (2002). Features of positive developmental settings, in NRCIM (Ed.), Community Programs to Promote Community Development. (Washington, DC: National Academy Press), 86–118.

[B13] Pierce D. Stas J. Feller K. Knox W. (2020). COVID-19: return to youth sports. Sports Innov. J. 1, 62–80. 10.18060/24144

[B14] Power M. Doherty B. Pybus K. Pickett K. (2020). How Covid-19 has exposed inequalities in the UK food system: the case of UK food and poverty. Emerald Open Res. 2, 1–22. 10.35241/emeraldopenres.13539.2

[B15] Project Play (2020). Survey: Parents Grow More Worried About Their Child Returning to Sports. Retrieved from: https://www.aspenprojectplay.org/coronavirus-and-youth-sports/reports/2020/7/14/survey-parents-grow-more-worried-about-their-child-returning-to-sports (accessed July 15, 2020).

[B16] Vest Ettekal A. Agans J. P. (2020). Positive youth development through leisure: confronting the COVID-19 pandemic. J. Youth Dev. 15, 1–20. 10.5195/jyd.2020.962

[B17] WSN (2020). UW Health Research Study Results Show Significant and Alarming Mental Health Impacts on School Closures and Sport Cancellations. Retrieved from: https://www.wissports.net/news_article/show/1110971 (accessed July 15, 2020).

